# Supplementary material for: Saccharomyces boulardii (CNCM I-745) improves intestinal damage in sepsis by remodeling intestinal flora structure
Source: Front Cell Infect Microbiol. 2025 Jul 31;15:1602792. doi: 10.3389/fcimb.2025.1602792 (PMC12350329; doi:10.3389/fcimb.2025.1602792)
Supplement: Supplementary file 1 [file DataSheet1.zip › summary/summary/7_Differential_analysis/1_diff_abundance/6_Genus/Group.CW4_vs_AW4/Group.CW4_vs_AW4_diff_barplot.pdf]

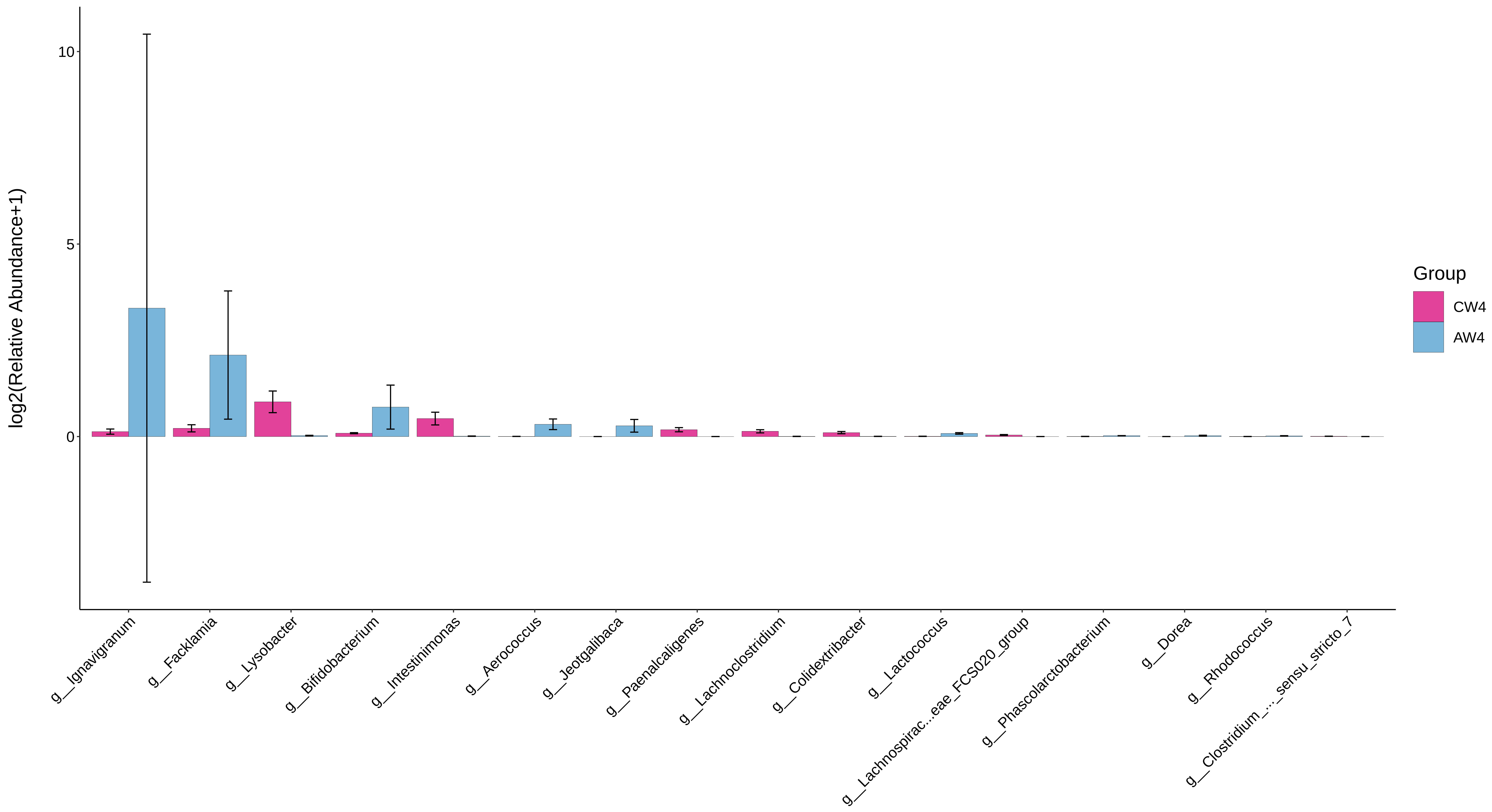
log2(Relative Abundance+1)
10
5
0
g__Ignavigranum
g__Facklamia
g__Lysobacter
g__Bifidobacterium
g__Intestinimonas
g__Aerococcus
g__Jeotgalibaca
g__Paenalcaligenes
g__Lachnoclostridium
g__Colidextribacter
g__Lactococcus
g__Lachnospirac...eae_FCS020_group
g__Phascolarctobacterium
g__Dorea
g__Rhodococcus
g__Clostridium_..._sensu_stricto_7
Group
CW4
AW4
